# Radiation Enterocolitis Featuring the Perforation of the Sigmoid Colon, Small Bowel, and Entero-Colonic Fistula: A Case Report

**DOI:** 10.7759/cureus.43167

**Published:** 2023-08-08

**Authors:** Muzi Meng, Diego Hanssen, Ajit Singh

**Affiliations:** 1 School of Medicine, American University of the Caribbean, Miramar, USA; 2 General Surgery, BronxCare Health System, Bronx, USA; 3 Surgery, Moffitt Cancer Center, Tampa, USA

**Keywords:** enterocolitis, surgery general, sigmoid perforation, entero-colonic fistula, radiation enterocolitis

## Abstract

Radiation enteritis poses a treatment challenge for patients undergoing or completing radiation therapy. A significant issue has been the patient's and surgeon’s lack of awareness of the condition and the radiotherapy or associated surgical treatments. A 66-year-old female presented with acute onset of diffuse abdominal pain and peritonitis for one day, status post radiation therapy following a diagnosis of cervical cancer. A review of systems was positive for diffuse sweating, chills, and nausea. The patient was diagnosed with an entero-colonic fistula with mesenteric edema. An entero-colonic fistula due to radiation enterocolitis is a rare but important complication that can occur after radiation therapy for abdominal or pelvic malignancies. With any patient who has a history of abdominal or pelvic cancer and has received radiotherapy and shows up with acute abdomen, bowel perforation should be considered in the differential diagnosis with the possible management of acute complications.

## Introduction

Radiation enteritis

Radiation enteritis (RE) is the inflammation of the intestine as a result of exposure to ionizing radiation therapy. It is a notable risk for patients undergoing treatment for tumors of the pelvic and abdominal area [1]. Despite being recognized as early as the 1950s, the condition has continuously remained poorly understood [2]. Presenting in both acute and chronic forms, initially thought to be rare, the condition’s incidence is believed to be on the rise, associated with an increasing incidence of abdominal and pelvic cancer [3]. RE can have a high mortality rate regardless of the success of the initial radiation therapy [4]. Risk factor modification that is patient and treatment-related is essential for prevention. The condition is a significant clinical problem that needs to increase awareness among patients and physicians.

Radiation enterocolitis

Radiation enteritis and radiation enterocolitis (RC) are related conditions but refer to slightly different aspects of radiation-induced intestinal damage. RE specifically refers to inflammation and damage to the small intestine due to radiation therapy [5]. A high cell turnover rate generally characterizes the small intestine, making it particularly susceptible to radiation [6]. Based on such features, RE usually leads to damage affecting the lining of the small intestine and can cause symptoms like abdominal pain, diarrhea, and weight loss. In contrast, RC refers to harm to the colon, that is, the large intestine, and includes inflammation and harm brought on by radiation therapy [7].

Harb et al. noted that acute RE occurs within hours to days after radiation therapy while chronic changes could occur between two months to 30 years after radiation therapy [8]. In RC, the inflammatory process often extends beyond the small intestine and involves the colon, causing symptoms similar to RE, including abdominal pain, diarrhea, and potentially rectal bleeding [9]. Nguyen et al. noted that RE and RC usage may differ among medical practitioners and researchers [10]; however, the terms are occasionally used interchangeably. They were collectively known as radiation-induced enteropathy; the disorder, whether RE or RC, is brought on by radiation therapy that results in intestinal inflammation and damage. Patients who undergo radiation therapy for tumors of the pelvis or abdomen, such as colorectal, prostate, or gynecologic cancers, are frequently affected [11]. Although radiation therapy is a successful cancer treatment, it can potentially harm nearby healthy tissues. As a result, RE can result from radiation beams accidentally damaging the cells lining the intestines when radiation is directed at the abdominal or pelvic region. The radiation dose, the length of therapy, the size of the treatment region, and the patient's radiation sensitivity are some variables that affect the severity of the condition [7].

Common symptoms include abdominal pain, diarrhea, nausea, vomiting, weight loss, and bowel obstruction. In addition, RE can, in severe cases, produce intestinal scarring and constriction, which can result in bowel blockage. As a result, one may experience excruciating stomach pain, bloating, and difficulty passing gas or stool. Therefore, it is crucial for people who have received radiation therapy to inform their healthcare professionals of any symptoms they have. Additionally, RE can have a minimally detrimental effect on a patient's general health with early discovery and adequate management [2].

Symptoms of RE can vary and typically develop weeks to months after the completion of radiation therapy. Short small bowel stricture represents the typical image of post-radiation therapy RE [2]. In contrast, RC involving the colon may show up in CT as hardening of the large intestine [12].

## Case presentation

A 66-year-old female with a past medical history of atrial fibrillation on Eliquis, hypertension, abdominal aortic aneurysm, cervical cancer (diagnosed the year prior status post radiation therapy internal and external for six weeks) open heart surgery for valve replacement, presented with a day's history of diffuse abdominal pain and peritonitis with diffuse sweating, chills, and nausea.

Clinical examination revealed diffuse severe tenderness to palpation, with rebounds and voluntary and involuntary guarding in the abdomen. Laboratory examination showed WBC (neutrophils) of 6.3, hemoglobin of 7.8, blood urea nitrogen (BUN)/creatinine (Cr) of 16/1.6, Liver function test (LFT) within normal limits (WNL), and lactic acid of 2.0.

**Figure 1:**
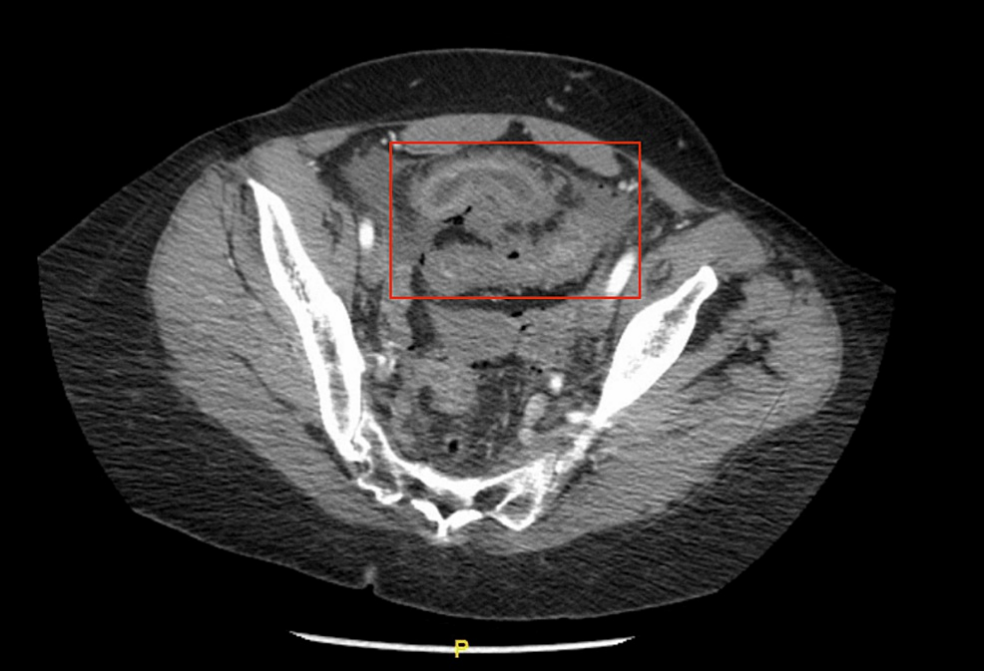
CT Angiography/Abdomen and Pelvis With and Without Contrast Axial View The abdomen and pelvis CT scan revealed the presence of free air and a fistula between the small bowel and the sigmoid colon (red box).

**Figure 2:**
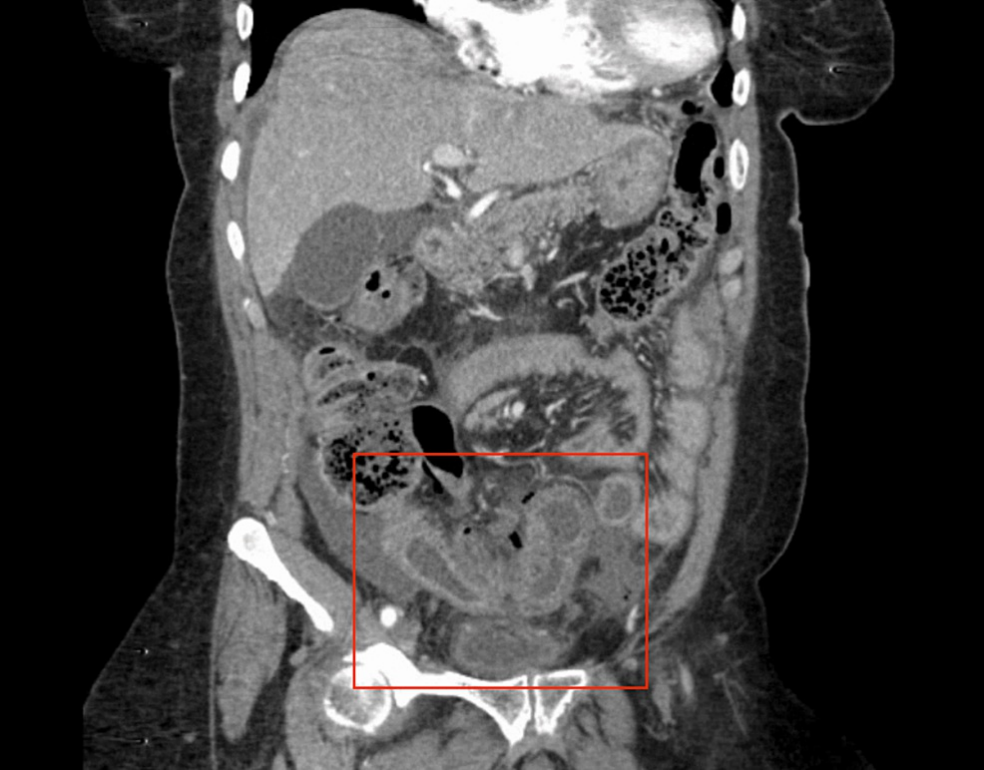
CT Angiography/Abdomen and Pelvis With and Without Contrast Coronal View The abdomen and pelvis CT scan revealed an inflammatory adhesion between the small bowel and sigmoid colon (red box).

Upon subsequent workup in the emergency department, a CT scan of the abdomen and pelvis showed free air in the peritoneum, a questionable connection between the small bowel and sigmoid colon (Figure 1), and inflammatory adhesions (Figure 2).

Pertinent imaging showed an impression of perforated enteritis with an entero-colonic fistula. Small bowel wall thickening was present in the lower abdomen. Mesenteric edema, mild ascites, and mild to moderate free air are associated. However, a fistula between the thick-walled bowel segments and the mid-sigmoid colon was apparent. The patient underwent exploratory laparotomy and during inspection of the bowel about 50 cm proximal to the cecum, there was an area of thickened and dilated bowel with a fistula between the sigmoid colon and the small bowel, associated with a 1 cm perforation. About 20 cm of small bowel were resected with primary anastomosis, and about 7 cm of sigmoid was resected with a fistulous portion and end colostomy was created. 

The small bowel specimen, small bowel staple line, and sigmoid colon specimen were sent for pathology review. Grossly, there was hemorrhagic serosa with fibrin adherence and exudate attached by about 1.5 cm mesenteric fat with an opening revealing gray-tan hemorrhagic mucosa with a transmural defect located 1 cm from the closest resection margin.

According to the pathology report, the small bowel showed a transmural perforation with acute inflammatory fibrinous exudate and acute serositis. The resection margins were viable with acute serositis and subserosal, edema, and chronic inflammation. The sigmoid colon showed transmural perforation with acute inflammatory fibrinous exudate and acute serositis.

## Discussion

Due to the increasing use of radiotherapy for pelvic cancers, chronic RC, a severe irradiation consequence, has gained attention [3]. Most reviews have shown RE to be eventually characterized by strictures and chronic malabsorption, which are all threatening complications and impact patient quality of life [2]. However, another consequence of pelvic radiation is the fistula [13], namely, a connection between two epithelial surfaces that is abnormal. Fistulas are named after the two lumens or surfaces they connect [14]. The complications are identified as one of those disorders found in patients who have undergone radiotherapy [15]. Different reported cases with multivariable analysis and emergency operation have also shown related cases of anastomotic fistula as independent risk factors for relapses in many male surgical procedures [16].

Such a radiation-induced fistula is typically initially screened with CT and then MRI in certain circumstances. Endoscopy can also be used to evaluate mucosal surfaces; it can provide information about the patient’s ongoing malignancy situation. In the cases reported, the CT abdomen and pelvis (AP) has shown the entero-colonic fistula to be associated with mesenteric edema with free air in the abdomen [17]. The fistula was also apparent between the thick-walled bowel segments and the mid-sigmoid colon of the patient. Regarding the pathophysiologic mechanism of the segment, the features were noted to lead to chronic intestinal inflammation, which further induces mucosal ulceration and eventual strictures with perforation, an abscess, and/or the entero-colonic fistula. The issue with such pathological changes is the possibility of causing chronic obstruction and microbial overgrowth in a life-threatening state.

Fistulas from radiation exposure are typically challenging to manage and heal [18]. They can manifest after a long lag period and will typically involve chronic inflammation. Fistulas can even arise many years after radiation therapy. While studies have found that the bulk of fistulas will occur within two years of radiation therapy, such as Gilinsky et al.’s study of 88 patients in the UK, Iwamuro et al. wrote of a case of fistula occurring 35 years after radiation exposure [19]. The latter note that fistulas tend to arise even after an extensive delay because pelvic radiation permanently alters the healing process due to poor tissue oxygenation and perfusion [19].

Surgery remains the best practice indicated treatment for managing the patient with fistulas such as the entero-enteral fistula caused by chronic RE [20]. Given the extent of inflammation that can arise in RE and RC, newly diagnosed fistulas may need special imaging and some delay before operating. This is because there is a risk of causing further injuries. The typical surgical approach is bowel segment excision. However, if there is malignancy, more radical excision is needed [15].

Risk factors that are both patient and treatment-related need to be appropriately reviewed in patients receiving radiotherapy treatment and those identified need to be modified where possible.

With any patient who has a history of abdominal or pelvic cancer and has received radiotherapy and who shows up with acute abdomen, bowel perforation should be considered in the differential diagnosis and possible management of acute complications.

## Conclusions

An entero-colonic fistula due to radiation enterocolitis is a rare but important complication that can occur after radiation therapy for abdominal or pelvic malignancies. This condition involves the formation of abnormal connections or channels between different segments of the intestine, resulting in considerable morbidity and reduced quality of life in affected individuals. Diagnosing an enteric fistula in radiation enteritis requires a high degree of suspicion because it can present with nonspecific symptoms such as abdominal pain, diarrhea, and weight loss. Radiographic imaging, including CT and contrast studies, plays a key role in identifying the fistula and determining its location and extent. In conclusion, entero-colonic fistulas caused by radiation enterocolitis are complex disorders that require a comprehensive approach, including accurate diagnosis, targeted treatment plans, and long-term follow-up. With any patient who has a history of abdominal or pelvic cancer and has received radiotherapy and shows up with acute abdomen, bowel perforation should be considered in the differential diagnosis and possible management of acute complications. Continued research is necessary to better understand the underlying mechanisms, identify risk factors, and develop more effective prevention and treatment strategies.
